# Early postoperative intraperitoneal chemotherapy for lower gastrointestinal neoplasms with peritoneal metastasis: a systematic review and critical analysis

**DOI:** 10.1515/pp-2019-0007

**Published:** 2019-10-04

**Authors:** Mikael L. Soucisse, Winston Liauw, Gabrielle Hicks, David L. Morris

**Affiliations:** Department of Surgery, University of New South Wales, St George Hospital, Kogarah, New South Wales, Australia; Department of Surgery, Hôpital Maisonneuve-Rosemont, Université de Montréal, Montreal, Quebec, Canada; Department of Medical Oncology, University of New South Wales, St George Hospital, Kogarah, New South Wales, Australia

**Keywords:** appendiceal cancer, colon cancer, EPIC, HIPEC, peritoneal carcinomatosis, peritonectomy

## Abstract

**Background:**

Early postoperative intraperitoneal chemotherapy (EPIC) can be used in combination with cytoreductive surgery (CRS) and hyperthermic intraperitoneal chemotherapy (HIPEC) to treat patients with peritoneal carcinomatosis (PC) of multiple origins. The present study is a systematic review to evaluate the role of EPIC after CRS + HIPEC for appendiceal and colorectal cancers with PC.

**Content:**

We conducted a systematic search in PubMed according to the PRISMA guidelines and included all studies published before June 27 of 2019 comparing EPIC to HIPEC or the combination of both. Our search found 79 articles. After excluding non-relevant articles, a total of 13 retrospective clinical studies reporting on the efficacy and safety of EPIC compared to HIPEC or as a combination therapy for lower gastrointestinal neoplasms were analyzed. Initial EPIC reports led to its declined usage because of concerns with increased postoperative morbidity and uncertain added benefit on survival. Recent retrospective studies have been promising, showing significant improvements in OS and fewer issues with complications when adding EPIC to CRS + HIPEC.

**Conclusions:**

Current evidence is entirely retrospective and is conflicting. It is hoped that ongoing clinical trials and additional studies will clarify EPIC’s role in the treatment of patients with PC.

## Introduction

Peritoneal spread of advanced neoplasms arising from the gastrointestinal and gynecological systems are some of the most challenging cases to manage in surgical oncology. Because of the poor blood supply of the peritoneum and the abundance of viscera in the abdomen, this disease responds poorly to conventional chemotherapy and radiotherapy [1]. When added to other classical treatment modalities, CRS and intraperitoneal chemotherapy dramatically changed the regional management of peritoneal disease of multiple origins [2]. CRS consists of removing all visible tumor nodules, including diseased organs from the abdominal cavity and is usually followed by intraperitoneal chemotherapy to target microscopic residual tumor cells. This strategy allows the local administration of higher concentrations of chemotherapy agents while decreasing their multiple systemic toxicities [3].

There are many ways of effectively delivering chemotherapy in the peritoneum perioperatively and several techniques have been refined over time [4]. Since the 1990s, CRS with either HIPEC or normothermic EPIC have been developed to treat PC mainly for advanced colorectal and appendiceal tumors [5, 6]. Heating the intraperitoneal solution to 41–43 °C can have a direct cytotoxic effects on tumor [7] while enhancing the effectiveness of chemotherapeutic agents [8].

In the recent years, EPIC has fallen out of favor because of concerns about increased postoperative morbidity [9, 10, 11, 12, 13], superiority of HIPEC over EPIC in terms of survival [14, 15, 16, 17] and because of the increased resources needed to manage EPIC in the ICU or on the ward. Undoubtedly, HIPEC is the current standard for the delivery of intraperitoneal therapy. This article aims to review the whole body of literature surrounding the use of EPIC for lower gastrointestinal neoplasms with PC. Most recent publications from our group and others, suggest that EPIC may have a survival advantage when added to HIPEC, without increasing postoperative complications [18, 19, 20, 21, 22, 23]

## Methods

A systematic PubMed database search was conducted on the 27th of June 2019 using the following keywords: ((((((early[All Fields] AND (“postoperative period”[MeSH Terms] OR (“postoperative”[All Fields] AND “period”[All Fields]) OR “postoperative period”[All Fields] OR “postoperative”[All Fields]) AND intraperitoneal[All Fields] AND (“drug therapy” [Subheading] OR (“drug”[All Fields] AND “therapy”[All Fields]) OR “drug therapy”[All Fields] OR “chemotherapy” [All Fields] OR “drug therapy”[MeSH Terms] OR (“drug”[All Fields] AND “therapy”[All Fields]) OR “chemotherapy” [All Fields])) AND (“peritoneal neoplasms” [MeSH Terms] OR (“peritoneal”[All Fields] AND “neoplasms”[All Fields]) OR “peritoneal neoplasms”[All Fields] OR (“peritoneal” [All Fields] AND “carcinomatosis”[All Fields]) OR “peritoneal carcinomatosis” [All Fields])) AND English[Language]) NOT ovarian[All Fields]) NOT (“stomach”[MeSH Terms] OR “stomach”[All Fields] OR “gastric”[All Fields])) NOT (“mesothelioma”[MeSH Terms] OR “mesothelioma” [All Fields])) NOT review[Publication Type] AND “humans” [MeSH Terms].

**Figure 1:**
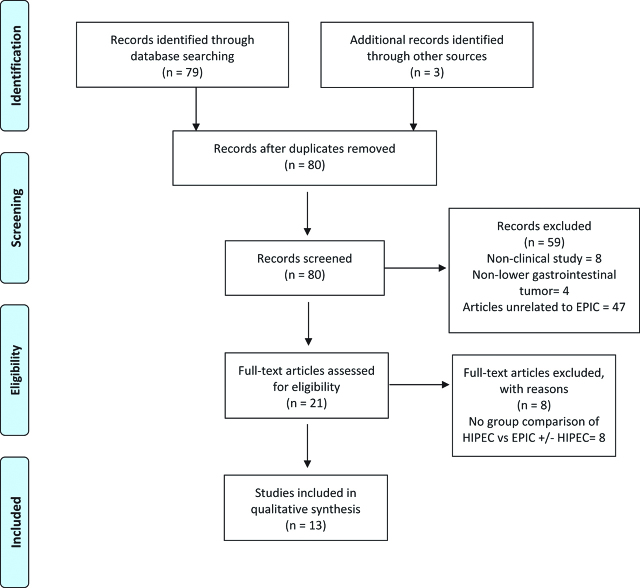
PRISMA flow diagram for this systematic review.

The search yielded 79 studies and 3 more were identified by screening previous systematic reviews on the use of intraperitoneal chemotherapy for lower gastrointestinal tumors with peritoneal metastases. The PRIMA Flow diagram is presented in Figure 1. Clinical studies containing patients who received EPIC after CRS for PC of appendiceal or colorectal origin were included in our review. We reviewed in detail a total of 21 clinical studies and excluded 8 additional studies because they were lacking group comparisons of HIPEC vs. EPIC ± HIPEC.

## Results

Based on inclusion and exclusions criteria, a total of 13 studies were analyzed and are detailed in Tables 1 and 2. All studies were retrospective cohort or case control studies with high risk of selection bias. There are currently no prospective randomized published studies looking at EPIC for PC of appendiceal and colorectal origin. Most of the studies were small and non-powered but there were three large international cohort studies [9, 22, 25] less likely to be underpowered. One must be cautious when interpreting Tables 1 and 2 as individual patients are likely to be represented in multiple studies, contributing to both national and international series. Reporting of morbidity and survival differed significantly between studies, making comparisons tenuous. Nevertheless, 9 out of 13 studies found that EPIC was associated with increased morbidity compared to HIPEC alone or as a dual therapy. Only 4 of those were statistically significant differences. Out of the 7 studies comparing HIPEC to HIPEC + EPIC that reported on survival, 5 showed a survival advantage with the combination therapy. Only the two most recent studies from our group were statistically significant [19, 20].

**Table 1:** Studies comparing HIPEC to EPIC for lower gastrointestinal tumors with peritoneal metastasis following cytoreductive surgery.

Author, year, country	Origin	n=	Treatment regimen	Grade 3 + morbidity	Survival analysis
Elias [14] France	Colorectal	23 23	HIPEC EPIC	0 fistula p=0.0216 6 fistulas	54% 5Y OS. NS 28% 5Y OS
Sideris [24]	Appendix	11 13	HIPEC EPIC	Not reported	60% 5Y OS. NS 58% 5Y OS
Elias [25]International	Colorectal	439 84	HIPEC EPIC	No difference	26% 5Y OS. NS 30% 5Y OS
Sorensen [16]Norway	PMP	45 48	HIPEC EPIC	17% NS 29%	79% 7Y OS. NS 75% 7Y OS

HIPEC, Hyperthermic intraperitoneal chemotherapy; EPIC, Early postoperative intraperitoneal chemotherapy; NS, Non statistically significant; PMP, pseudomyxoma peritonii; OS Overall survival.

**Table 2:** Studies comparing HIPEC to the combination of HIPEC + EPIC for lower gastrointestinal tumors with peritoneal metastasis following cytoreductive surgery.

Author, year, country	Origin	n=	Treatment regimen	Grade 3 + morbidity	Survival analysis
Glehen [9]International	Colorectal	271 112	HIPEC HIPEC + EPIC	EPIC=more fistulasRR 1.7. p=0.032	HIPEC + EPIC Better than HIPEC and EPIC alone but NS p=0.61
Saxena [26]Australia	Colorectal	12 34	HIPEC HIPEC + EPIC	50% NS 30%	Not reported
Chua [22]international	PMP	1382 668	HIPEC HIPEC + EPIC	No difference	HIPEC found to be an independent factor of better OS but not EPIC
Chua [21]Australia	Colorectal Subgroup^a^	30 45	HIPEC HIPEC + EPIC	13% 16%	19 months RFS. 19 months OS 33 months RFS. 38 months OS p=0.046. p=0.38
Lam [15] Canada	Colorectal + High grade appendix	37 56	HIPEC HIPEC + EPIC	19.6% p=0.01 43.2%	6% 3Y RFS 46% 3Y OS. 21% 3Y RFS 50% 3Y OS. NS. NS
Sparks [13]Australia	Appendix	13 17	HIPEC HIPEC + EPIC	Trend toward more complications with EPIC group NS	No difference
Tan [12]Singapore	multiple	69 42	HIPEC HIPEC + EPIC	25% p=0.048 58%	Not reported
Huang [19]Australia	LAMN	74 176	HIPEC HIPEC + EPIC	44.6% 48.3%	64.5% 5Y OS. p=0.001 93.0% 5Y OS
Huang [20]Australia	PMCA	118 67	HIPEC + EPIC	47.9% 53.7%	30.5% 5Y OS. p=0.003 62.3% 5Y OS

a Appendiceal neoplasms reported in later case control studies of the same unit [19, 20].

HIPEC, Hyperthermic intraperitoneal chemotherapy; EPIC, Early postoperative intraperitoneal chemotherapy; NS, Non statistically significant; PMP, pseudomyxoma peritonii; OS Overall survival; RFS, Recurrence free survival; LAMN, Low-grade appendiceal mucinous neoplasm; PMCA, Peritoneal mucinous carcinomatosis of the Appendix.

## Summary of evidence – foundation of EPIC

EPIC was first introduced by Sugarbaker in the 1990s in an effort to reduce disease recurrence and to prolong long-term survival of patients with PC [27]. Given the high risk of peritoneal recurrence, even after optimal cytoreduction, EPIC was a simple way of delivering high doses of cytotoxic agents targeted at the peritoneal surfaces without systemic compromise [28]. Sugarbaker’s design consisted of administering dilute 5-FU through a Tenckhoff catheter on postoperative days 1 to 5 in order to eliminate any residual microscopic tumor deposits before the formation of fibrinous adhesions [29]. The most commonly used protocol for appendiceal and colorectal neoplasms is 650  mg/m^2^ of 5-FU infused in hypertonic, high molecular weight solution to reduce its clearance speed from the abdominal cavity [30]. This solution is infused for 23 h with the surgical drains clamped, followed by 1 h of free drainage. This protocol overcomes the disadvantage of 5-FU’s short half-life because of its high intraperitoneal/intravenous area under the curve ratio, which allows the administration of higher doses with a resultant 250-fold increased tissue exposure [31]. Even with very high doses of 5-FU, systemic toxicities are much lower than systemic infusion because of the first pass metabolism through the liver. 5-Fu toxicity can be markedly increased in dihidropyrimidine dehydrogenase (DPD) deficient subjects, which represents between 3% and 15% of the population. Most cancer associations now recommend systematic screening for DPD deficiency before initiating any 5-FU based therapy [32]. In a murine experimental study by Klaver et al., comparing CRS alone vs. CRS + HIPEC vs. CRS + EPIC vs. CRS + HIPEC + EPIC, both EPIC and HIPEC were shown to prolong the rat’s survival [33]. A 1:2 matched case-control study comparing EPIC (n=30) vs. CRS only (n=15) for colorectal cancers identified EPIC as an independent prognostic factor for both OS and DFS [34]. This is one of the few clinical studies to have studied EPIC’s standalone efficacy when added to an optimal CRS for lower gastrointestinal tumors. Armstrong et al. reported in 2006 the results of an RCT which demonstrated significantly better OS with EPIC vs. IV chemotherapy after optimal CRS for ovarian PC (23.8 vs. 18.3 months respectively) [35]. Another RCT published in 2001 demonstrated similar results in favor of adding EPIC after radical surgery for locally advanced gastric cancers [36]. Multiple studies have compared HIPEC and EPIC head to head after CRS and all have found HIPEC to be either equivalent or superior in terms of survival as shown in Table 1 [14, 16, 24, 25]. A retrospective matched case control study by Elias et al. of 46 patients found that HIPEC was associated with a better 5-year OS compared to EPIC (54% vs. 28%), although not statistically significant [14]. Concerningly, patients in the EPIC group had more postoperative fistulas, more peritoneal recurrences and worse long-term survival compared to patients who received HIPEC. In 2003, Verwaal *et al.* published the first randomized controlled study using HIPEC for PC of colorectal origin [37]. This trial demonstrated a significant increase in OS with the use of CRS + HIPEC compared to systemic therapy alone. Being the only published RCT for PC at that time, this trial set CRS + HIPEC as the new standard for the treatment of selected appendiceal and colorectal neoplasm with PC.

## Summary of evidence HIPEC + EPIC combination therapy

After 2003, many units around the world continued to use Sugarbaker’s initial protocol consisting of Mitomycin C (MMC) HIPEC and 5-FU EPIC. In 2012, Chua *et al.* conducted a large multicenter retrospective study of 2298 patients with PMP of appendiceal origin treated with CRS + HIPEC + EPIC [22]. In this study, although EPIC was significantly associated with longer OS in the univariate analysis, this effect was no longer present in the multivariate analysis, which could mean that the use of EPIC is more of a surrogate for another factor which positively influences OS rather than an independent factor.

Our group reported on a retrospective cohort study comparing different intraperitoneal chemotherapy regimens for PC of colorectal and appendiceal origin and found no significant impact of adding EPIC to HIPEC on OS [21]. In this cohort, the addition of EPIC to HIPEC was associated with longer recurrence free survival (33 vs. 19 months p=0.046) only in patients with PC of colorectal origin. These results have to be interpreted with caution as there was a significant risk of selection bias in the study design. The standard intended treatment in our unit at that time was HIPEC + EPIC and the control group consisted of patients who were not eligible to receive EPIC. In circumstances where high risk surgical procedures were performed and with reasonable risk of complications, leakage of intraperitoneal chemotherapy, major organ failure, intra-abdominal hypertension and hemodynamic instability, EPIC was withheld.

Lam *et al.* published the Canadian’s experience with EPIC in 2015 for the treatment of PC of appendiceal and colorectal origin. After reports of increased complications with the use of EPIC [9, 14], this unit changed their intraperitoneal chemotherapy protocol in 2008. Initially, their protocol was HIPEC with MMC (12–15 mg for 60 min) followed by 5-FU EPIC (1000  mg daily from post-operative day 1 to 5). After 2008, the protocol was changed to HIPEC with 400 mg of oxaliplatin for 60 min with a simultaneous IV dose of 5-FU, but no further EPIC was given. This particular historical context provided the opportunity to examine differences in complications and survival without significant selection bias. The authors first reported the impact of the different regimen on major complications and found that the combination of HIPEC + EPIC and PCI>26 were the only two independent factors associated with grade 3 + morbidity [10]. The concern for a learning curve effect with this study design was addressed by analysing overall major complications according to the year of surgery without finding any trend. Two years later, they reported their survival data of the same historical cohort and found no significant difference in the 3-year OS [15]. The two groups were very similar but more chemotherapy was given to the HIPEC group (76.8% vs. 54% p=0.05). The 60-minute fixed 12–15 mg dose of MMC used in the HIPEC + EPIC group is one of the lowest doses reported. Doses between 20 and 40  mg of MMC for 90 min are commonly seen in large cohort studies. These two factors should be considered when analysing the survival of the two groups, because they both favor the HIPEC only group. The authors reported no difference in RFS but when analysing the data, the 3-year RFS was 21% in the HIPEC + EPIC group compared to 6% in HIPEC group. Although not statistically significant, there is still an observable difference in RFS between the two groups in favor of HIPEC + EPIC.

In 2016, the Singapore group also reported their data which were consistent with previously published studies [12]. In this retrospective study of 111 subjects with tumors of different origins, patients all received HIPEC but did not get EPIC if they underwent a very extensive surgery. In fact, the no EPIC group had significantly more blood losses, longer ICU stay and more transfusions than the EPIC group. Despite this, patients with less extensive surgery who receive EPIC still presented more grade 3 and above complications (58% vs. 25%) and a longer hospital stay (16 vs. 13 days). Survival analysis pointed toward better OS in favor of the combined therapy with an HR of 0.62 (0.28–1.37, p=0.231).

In the past two years, new evidence arising mainly from our center in Sydney Australia has emerged and differs from previous findings, reopening a debate that many thought was closed. EPIC or no EPIC?

In 2017, Huang *et al.* published two papers on our experience with EPIC for Low grade appendiceal mucinous neoplasms (LAMN) [20] and appendiceal adenocarcinomas (PMCA) [19].

In the first study, 250 consecutive surgeries for LAMNs were retrospectively analyzed to compare HIPEC vs. HIPEC + EPIC. The two groups were generally comparable but the HIPEC group was on average 4 years older, putting them more at risk than the combined therapy group. No difference in postoperative mortality or morbidity was found and patients who received EPIC had a significantly better 5-year OS (93.0% vs. 64.5% p=0.001). When comparing the five year OS of both groups to a large retrospective multicenter cohort study [22], LAMNS who received HIPEC + EPIC seem to have comparable 5-year OS (93% vs. 91%) whereas patients receiving HIPEC only had a worse prognosis than what is quoted in the literature (64% vs. 76%), despite excluding CCR-2 patients, which were included in the large cohort study. With the two cohorts having similar mean PCI (22 and 21) and median age (53 and 53), it seems that the 5-year OS difference identified in this study is related to the HIPEC only group having a worse prognosis, raising the suspicion for selection bias, although we cannot draw conclusions from comparing the two study cohorts. In fact, as we noted in the text, the default treatment in our unit for soft or LAMN appearing tumors is HIPEC + EPIC and the decision to withhold EPIC is either subjective or because of contraindications to EPIC. The second study [19] with a similar retrospective case-control design (HIPEC + EPIC vs. HIPEC only), focused on patients with PMCAs. The groups differed significantly in terms of chemotherapy agent used for the HIPEC, MMC was used 95.5% of the time in the HIPEC + EPIC group whereas Oxaliplatin was used in 76% of the time in the HIPEC only group. There was no significant difference in terms of hospital mortality (p=0.632), major morbidity rate (i.e. Grade III/IV) (p=0.444), ICU stay (p=0.638) and total hospital stay (p=0.078). Patients who received HIPEC and EPIC had a significantly better 5-year OS than those who received HIPEC alone (62.3% for HIPEC + EPIC, 30.5% for HIPEC alone, p=0.002) which reflected on better PFS as well (18.0 vs. 12.3 months, p=0.002). Although there was a significant difference in the type of chemotherapy agent used in the two groups, Levine *et al.* published an RCT in 2018 demonstrating no significant survival advantage between MMC and Oxaliplatin for appendiceal tumors with PC treated by CRS + HIPEC [38].

We later published interesting findings on the correlation between subjective tumor consistency of PMCAs and long-term survival [23]. In this retrospective study of 192 patients, subjects with softer tumors tended to have received EPIC more commonly on top of HIPEC but when adjusting for EPIC in the multivariate analysis, subjective tumor consistency was found as an independent survival prognostic factor.

Analyzing the only two studies to have found such a positive impact of adding EPIC over HIPEC [19, 20], nearly doubling the survival of patients with LAMNs and PMCAs, one must suspect significant selective bias and it is now clear that choosing which patient receives EPIC according to tumor’s consistency is potentially one of them. After realizing the impact of tumor consistency on survival and the potential for bias, we did a post hoc analysis to adjust for tumor consistency in the multivariate analysis. After adjustments, EPIC remained an independent factor for better OS of soft (5Y OS 76.4% vs. 44.0%, p=0.005) and intermediate (p=0.06) tumors [23].

Interestingly, most recent published data do not seem to show increased morbidity with the use of EPIC compared to older series. Because of the retrospective nature of these studies, this could be due to selection bias but could also be due to better postoperative care in general.

## Discussion

Current available data on the efficacy of EPIC when combined with HIPEC for the treatment of PC of appendiceal and colorectal origin is conflicting and difficult to interpret due to the retrospective nature of all the studies and the possibility of bias. To date, there are no published prospective data regarding the use of EPIC for PC of appendiceal and colorectal origin which, combined with concerns of increased postoperative morbidity and inconsistent effects on survival, explains why EPIC has fallen out of favor over time. Although decreasing postoperative complications is something every surgeon seeks, survival and quality of life is really what matters most to cancer patients [39]. Specifically in the CRS and HIPEC literature, it is not clear if postoperative complications correlate with long term OS or not [22, 40, 41, 42].

All the studies to have compared HIPEC vs. HIPEC + EPIC are presented in Tables 1 and 2. Most studies reported increased morbidity with the combined therapy. Although most initial papers comparing HIPEC + EPIC vs. HIPEC did not find statistically significant survival differences, all pointed toward better survival with the combined therapy. Concluding there is no difference in survival because the p-value is not below 0.05 is a common mistake and is a false statement [43]. In such small samples, it is merely impossible to know if there is a Beta error or if no association exists. Although not exempt from bias, the fact that two recent studies have found clinically and statistically significant better survival with HIPEC + EPIC warrants further investigations.

ICARuS is an actively recruiting multicenter RCT in the US, evaluating the effectiveness of EPIC after optimal CRS + MMC HIPEC for patients with isolated peritoneal metastasis of appendiceal and colorectal origin (NCT01815359). This trial will certainly be very informative on the effectiveness of EPIC, but survival results won’t be available for many years. Additional propensity matched case control studies from other high-volume centers could potentially enlighten us in the meantime. Making a parallel with malignant peritoneal mesothelioma, Sugarbaker et al. recently published their long experience, comparing three different chemotherapy regimens (HIPEC vs. HIPEC + EPIC vs. HIPEC + EPIC + normothermic intraperitoneal chemotherapy long term (NIPEC)) [18]. No significant survival benefit was observed when adding EPIC over HIPEC, but interestingly, a long-term administration of paclitaxel (NIPEC) significantly increased the 5-year OS from 44 to 75%. As local recurrence and complications such as malignant bowel obstruction is still an important problem for patients with PC, further investigations on the use of EPIC and other postoperative intraperitoneal regimens are needed and should not be hampered by the fear of possibly increasing postoperative complications, if the resultant effect is better survival and quality of life for our patients.
